# Decreased histone deacetylase 2 impairs Nrf2 activation by oxidative stress

**DOI:** 10.1016/j.bbrc.2011.02.035

**Published:** 2011-03-11

**Authors:** Nicolas Mercado, Rajesh Thimmulappa, Catherine M.R. Thomas, Peter S. Fenwick, Kirandeep K. Chana, Louise E. Donnelly, Shyam Biswal, Kazuhiro Ito, Peter J. Barnes

**Affiliations:** a Airway Disease Section, National Heart and Lung Institute, Imperial College, London SW3 6LY, UK; b Department of Environmental Health Sciences, Johns Hopkins Bloomberg School of Public Health, Baltimore, MD, USA

**Keywords:** ARE, anti oxidant response element, COPD, chronic obstructive pulmonary disease, DJ-1, Parkinson’s disease (PD)-associated protein, HDAC2, histone deacetylase-2, HO-1, heme oxygenase-1, H_2_O_2_, hydrogen peroxide, Keap1, Kelch-like ECH associated protein 1, MDM, monocyte-derived macrophage, Nrf2, nuclear factor erythroid 2-related factor 2, ROS, reactive oxygen species, TSA, trichostatin A, Oxidative stress, Nrf2, Histone deacetylase 2, Protein stability, Acetylation, COPD

## Abstract

Nuclear factor erythroid 2-related factor 2 (Nrf2) plays a crucial role in cellular defence against oxidative stress by inducing the expression of multiple anti-oxidant genes. However, where high levels of oxidative stress are observed, such as chronic obstructive pulmonary disease (COPD), Nrf2 activity is reduced, although the molecular mechanism for this defect is uncertain. Here, we show that down-regulation of histone deacetylase (HDAC) 2 causes Nrf2 instability, resulting in reduced anti-oxidant gene expression and increase sensitivity to oxidative stress. Although Nrf2 protein was clearly stabilized after hydrogen peroxide (H_2_O_2_) stimulation in a bronchial epithelial cell line (BEAS2B), Nrf2 stability was decreased and Nrf2 acetylation increased in the presence of an HDAC inhibitor, trichostatin A (TSA). TSA also reduced Nrf2-regulated heme-oxygenase-1 (HO-1) expression in these cells, and this was confirmed in acute cigarette-smoke exposed mice *in vivo*. HDAC2 knock-down by RNA interference resulted in reduced H_2_O_2_-induced Nrf2 protein stability and activity in BEAS2B cells, whereas HDAC1 knockdown had no effect. Furthermore, monocyte-derived macrophages obtained from healthy volunteers (non-smokers and smokers) and COPD patients showed a significant correlation between HDAC2 expression and Nrf2 expression (*r* = 0.92, *p* < 0.0001). Thus, reduced HDAC2 activity in COPD may account for increased Nrf2 acetylation, reduced Nrf2 stability and impaired anti oxidant defences.

## Research highlights

► Nrf2 anti-oxidant function is impaired when HDAC activity is inhibited. ► HDAC inhibition decreases Nrf2 protein stability. ► HDAC2 is involved in reduced Nrf2 stability and both correlate in COPD samples. ► HDAC inhibition increases Nrf2 acetylation.

## 1 Introduction

Nrf2 is a redox-sensitive transcription factor that regulates the expression of phase II anti oxidant genes and confers cytoprotection against oxidative stress [1,2]. In unstressed cells Nrf2 is sequestered by its inhibitor, Keap1, that promotes rapid proteasome-mediated degradation via a Cul3 based E3 ubiquitin ligase complex [3]. However, in response to oxidative stress Nrf2 is stabilized by dissociating from Keap1, and binds to *cis*-elements called ‘‘antioxidant response elements” (ARE) as a heterodimer with other members of the basic leucine zipper protein family, such as Maf or Jun [4]. Nrf2 is localized mainly in the nucleus of airway epithelial cells and alveolar macrophages in lung and maintains anti-oxidant capacity to environmental or endogenous oxidative stress insults [5]. Persistent overload of reactive oxygen species (ROS) is reported to result in chronic inflammation in the lungs of patients with chronic obstructive pulmonary disease (COPD) [6]. In fact, Nrf2-deficient mice are highly sensitive to oxidative stress and develop severe emphysema to cigarette smoke [7,8]. Recently, whole lung tissue and alveolar macrophages from emphysema patients were reported to show decreased Nrf2 protein expression and activity and anti-oxidant genes due to an increase in the negative regulators Keap1 and Bach1 [9]. Another study on lung homogenates from patients with COPD found that defective Nrf2 expression was due to decreased expression of its positive regulator DJ-1 [10]. Peripheral lung and alveolar macrophages of COPD patients are also defective in histone deacetylase 2 (HDAC2), resulting in corticosteroid insensitive inflammation via hyper-acetylation of histones and glucocorticoid receptors [11,12]. Nrf2 has been recently found to be regulated by acetylation [13]. We therefore hypothesized that HDAC2 may control Nrf2 activity via deacetylation and that a decrease in HDAC2 may cause impaired function of Nrf2 leading to down-regulation of anti-oxidant responsive genes, such as HO-1.

## 2 Materials and methods

### 2.1 Cell culture, MDMs and stimulation

BEAS2B cells (human airway epithelial), (ATCC Teddington, UK) were cultured in keratinocyte media (Invitrogen, Paisley, UK) containing human recombinant epithelial growth factor (EGF) and bovine pituitary extracts (BPE). For mRNA stability experiments, BEAS2B cells were stimulated with actinomycin-D (Sigma, Poole, UK) (5 μM) and TSA (Sigma) (10 ng/ml) at the same time and incubated from 0 to 4 h. For protein stability experiments, BEAS2B cells were stimulated with cycloheximide (Sigma) (0.5 μg/ml) from 0 to 70 min in the presence of 30 min pre-incubation with TSA (50 ng/ml) and/or stimulation with H_2_O_2_ (50 μM) at the same time as cycloheximide.

Monocytes were isolated from peripheral blood mononuclear cells obtained from five healthy, three normal smoker volunteers and five COPD volunteers and differentiated into monocyte-derived macrophages [14]. Ethics approval was obtained from the Ethics Committee of the Royal Brompton & Harefield Hospitals National Health Service Trust, and all subjects gave written informed consent.

### 2.2 Nuclear and whole cell extraction

Nuclear extractions were performed using the Active Motif Nuclear Extraction kit (Rixensard, Belgium) following manufacturer’s instructions. Whole cell extracts were prepared using 0.5% (v/v) NP40 RIPA buffer as previously described [14].

### 2.3 Nrf2 activity assay

Cells were stimulated with H_2_O_2_ (75 μM) for different time points (0–24 h), with pre-treatment of TSA (0.1–1000 ng/ml) or MG132 (5 μg/ml) and nuclear extracts were used for the determination of Nrf2 binding activity to immobilized anti-oxidant response elements (ARE) using a TransAM^TM^ Nrf2 kit (Active Motif).

### 2.4 siRNA studies

BEAS2B cells were transfected with HDAC2 (10 nM) (Qiagen, Crawley, UK), HDAC1 (5 nM) (Dharmacon, Epsom, UK) or random oligonucleotide control (RO, 10 nM) siRNA (Qiagen) for 48 h using HiPerfect transfection reagent (Qiagen, Crawley, UK) following the manufacturer’s instructions.

### 2.5 Immunoprecipitation

Whole cell extracts from BEAS2B cells that were stimulated with MG132 (5 μg/ml, 2 h) and TSA (10 ng/ml, 1–2 h) were pre-cleared using rabbit polyclonal IgG and Protein A/G (Santa Cruz Biotechnology, Heildelberg, Germany) for 30 min. Supernatants were then incubated overnight with rabbit anti-Nrf2 (Santa Cruz) antibody at 4 °C and immunoprecipitation was performed as previously described [15].

### 2.6 Western blot

Proteins from whole-cell and nuclear extracts were separated by 10% SDS–PAGE (Invitrogen, Carlsbad, CA). Primary antibodies against Nrf2 (Rabbit, Santa Cruz and Mouse, R&D Systems), pan-acetylated lysine (Ac-K) (Bioscience, Cambridge, UK), β-actin (Abcam, Cambridge, UK), TBP (Abcam), HDAC1 (Sigma), HDAC2 (Sigma), Keap1, DJ-1 and Lamin A/C (Santa Cruz) were used to detect protein expression.

### 2.7 Animals and treatments

C57BL/6 mice (male, 8 week) were exposed to cigarette smoke for 5 h using a TE-10 smoke machine (Teague Enterprises, Davis, CA) and 2R4F reference cigarettes with a total suspended particle concentration of 250 mg/m^3^ as previously described [16]. One hour prior to cigarette smoke, mice were treated with vehicle and or TSA at a dose of 100 ug/ml in 50 μl of PBS intranasally. Immediately after cigarette smoke exposure, lungs were isolated and stored in fresh RNA later (Qiagen).

### 2.8 Real-time quantitative PCR

Total cellular RNA was extracted and cDNA was prepared as previously reported [11]. Real-time quantitative PCR (qRT-PCR) analysis of Nrf2 and GAPDH was performed using Taqman primer and probe set from Applied Biosystems (Warrington, UK), and HO-1 and GNB2L1 using Taqman primers and probe sets from Qiagen in the Corbett Rotor Gene 6000. For animal studies, qRT-PCR analyses was carried using Taqman primer probe set and reactions were analyzed using the ABI 7000 Taqman system as previously described [16].

### 2.9 Statistical analysis

Data are expressed as median ± SEM. Results were analyzed using t-test for hydrogen peroxide stimulation and one-way Anova for repeated measures with Dunnett post-test to determine the effect of various TSA concentrations. For MDMs, non-parametric Wilcoxon matched pairs was used to measure the effect of H_2_O_2_ or TSA. The Graph Pad Prism Software (Prism, San Diego, CA) was used for statistical calculations. *p* < 0.05 was considered statistically significant.

## 3 Results

### 3.1 Hydrogen peroxide increases Nrf2 protein levels and activity in BEAS2B cells

**Fig. 1:**
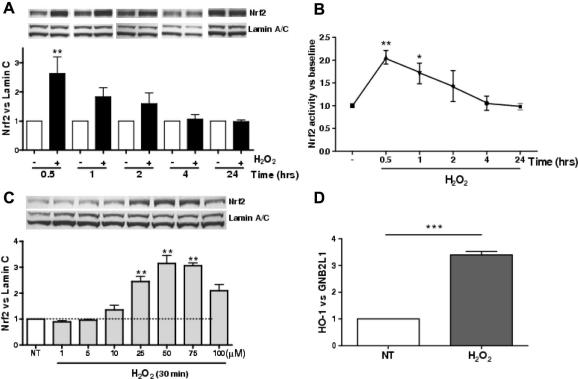
Hydrogen peroxide (H_2_O_2_) increases Nrf2 protein and activity in BEAS2B cells. (A) Cells were treated H_2_O_2_ (75 μM) for 0.5–24 h and nuclear Nrf2 was measured by Western blotting and normalized to the oxidant insensitive protein lamin C (*n* = 3). (B) The same nuclear extracts were used for measurement of Nrf2 DNA binding (*n* = 3). (C) Western blot analysis of BEAS2B cell nuclear extracts stimulated with H_2_O_2_ (1–100 μM) for 30 min (*n* = 3). (D) BEAS2B cells were stimulated with H_2_O_2_ (75 μM) for 8 h and HO-1 mRNA expression was normalized to GNB2L1 and analysed using qRT-PCR; NT: non-treatment. Values represent means ± SEM, ^∗^*p* < 0.05, ^∗∗^*p* < 0.01, ^∗∗∗^*p* < 0.005.

H_2_O_2_ (75 μM) increased Nrf2 protein levels as measured by Western blotting (Fig. 1A). Nuclear Nrf2 protein expression peaked 30 min after H_2_O_2_ treatment (2.6 ± 0.6-fold; *p* < 0.01) and gradually decreased to basal levels after 24 h. The same nuclear extracts were used to measure the binding ability of nuclear Nrf2 to ARE and showed the same pattern of early peak binding at 30 min (2.1 ± 0.2-fold; *p* < 0.01) (Fig. 1B). When cells were treated with different concentrations of H_2_O_2_ (1–100 μM) for 30 min, Nrf2 expression was maximal between 50 and 75 μM of H_2_O_2_. Expression of the oxidative stress responsive gene, HO-1, which confers cytoprotection against oxidative stress [17], was increased (3.4 ± 0.1-fold; *p* < 0.005) 8 h after H_2_O_2_ treatment (Fig. 1D).

### 3.2 HDAC inhibition reduces Nrf2 stability and impairs Nrf2 activation by oxidative stress

**Fig. 2:**
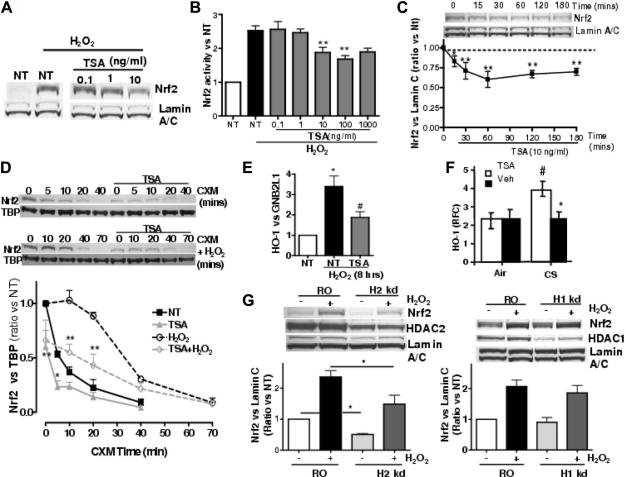
HDAC2 inhibition reduces Nrf2 protein stability. (A) BEAS2B cells were treated with TSA (0.1–10 ng/ml) for 1.5 h prior 30 min exposure to H_2_O_2_ (75 μM) and nuclear Nrf2 was measured by Western blotting. (B) Nrf2 DNA binding activity was measured in nuclear extracts stimulated with trichostatin A (TSA 0.1–1000 ng/ml) for 1.5 h prior to 30 min exposure to H_2_O_2_ (75 μM; *n* = 3; ^∗^*p* < 0.05, ^∗∗^*p* < 0.01 vs. H_2_O_2_). (C) Cells were stimulated with TSA (10 ng/ml) for 180 min and nuclear extracts analyzed for Nrf2 and lamin A/C expression by Western blotting. NT: non-treatment; *n* = 3. (D) Cells were stimulated with cycloheximide (CXM 0.5 μg/ml) in the presence or absence of 30 min pre-treatment with TSA (50 ng/ml). Cells were also stimulated with CXM in the presence of H_2_O_2_ (50 μM) with or without pre-treatment with TSA for 30 min. Nuclear extracts were analyzed by Western blot for Nrf2 and TBP as nuclear control. NT: non-treatment; *n* = 3. NT vs. TSA and H_2_O_2_ vs. H_2_O_2_ + TSA for each time point (^∗^*p* < 0.05, ^∗∗^*p* < 0.01). (E) Cells were treated with TSA (10 ng/ml) for 1.5 h prior to 8 h with H_2_O_2_ (75 μM). HO-1 expression was measured by QRT-PCR with GNB2L1 used as control (*n* = 3; ^∗^*p* < 0.05 between NT and H_2_O_2_, ^#^*p* < 0.05 between TSA and H_2_O_2_). (F) Mice were treated with intranasal TSA or vehicle (Veh) for 1 h prior to 5 h to cigarette smoke (CS) or air. HO-1 was measured against β-actin using QRT-PCR, (^#^*p* < 0.05 vs. Air/Veh, ^∗^*p* < 0.05 vs. CS/Veh, (*n* = 5/group)). (G) Cells were transfected with HDAC2 siRNA (H2 kd), HDAC1 siRNA (H1 kd) or a random oligonucleotide (RO) for 48 h and stimulated with H_2_O_2_ (50 μM) for 30 min. Nrf2, HDAC2, HDAC1 and Lamin A/C were measured by Western blot from whole cell extracts. Values represent means ± SEM, *n* = 4, ^∗^*p* < 0.05.

BEAS2B cells were treated with non-selective Class I and II HDAC inhibitor, TSA, to mimic the conditions seen in COPD [11]. As shown in Fig. 2B, TSA concentration-dependently inhibited Nrf2 activity in nuclear extracts, with maximum inhibition of 53% at 100 ng/ml. Nrf2 was not further inhibited at higher concentration of TSA (Nrf2 induction; 1.7 ± 0.1-fold a 100 ng/ml, 1.9 ± 0.2-fold at 10 ng/ml vs. H_2_O_2_: 2.5 ± 0.1-fold; *p* < 0.01). Thus, the inhibitory effect of TSA on the nuclear Nrf2 levels was significant but incomplete. In addition, when analyzing the same nuclear extracts by Western blot, Nrf2 protein levels were reduced at 10 ng/ml of TSA (Fig. 2A). The inhibition by TSA also happened in baseline Nrf2 and was detected 15 min after treatment, peaking at 60 min and lasting for 180 min (Fig. 2C). Although some reports indicate that Nrf2 is located in the cytoplasm [18,19], our experiments show that Nrf2 is detectable only within nuclei, before and after H_2_O_2_ treatment (Supplementary Fig. S1) and that TSA (10 ng/ml) prevents an increase of Nrf2 in the nuclei (Fig. 2B, Supplementary Fig. S1). Nrf2 protein half-life was measured by incubating cells in the presence of translational inhibitor cycloheximide (Fig. 2D). At baseline, Nrf2 was quickly degraded by the proteasome with a half-life of 6 min (*t*½ = 6 min) similar to other studies [18,20]. Hydrogen peroxide increased the stability of the protein (*t*½ = 33 min) and pre-incubation of TSA decreased baseline Nrf2 stability (*t*½ = 1.4 min) as well as H_2_O_2_-stimulated Nrf2 stability (*t*½ = 13.8 min) (Fig. 2D). The reduction of Nrf2 protein levels after TSA was not caused by decreased Nrf2 transcription or reduced mRNA half-life as shown by an experiment using actinomycin D (Supplementary Fig. S2A and B). Additionally, we tested the inhibition of Class III HDACs (sirtuins) using sirtinol, a non-specific sirtuin inhibitor and measured the effect of H_2_O_2_ on Nrf2 protein levels. Results show that increase in Nrf2 stability is not controlled by Sirtuins (Supplementary Fig. S2C). TSA also significantly inhibited H_2_O_2_-stimulated HO-1 expression (TSA: 1.9 ± 0.3-fold vs. H_2_O_2_: 3.4 ± 0.5-fold; *p* < 0.05, Fig. 2E).

### 3.3 HDAC inhibition impairs cigarette smoke-induced anti-oxidant response in mice *in vivo*

We performed an *in vivo* experiment to confirm the effects of HDAC inhibition on HO-1 expression observed *in vitro*. Mice were exposed to cigarette smoke for 5 h, and pulmonary HO-1 mRNA expression determined. As shown in Fig. 2F, cigarette smoke significantly increased HO-1 mRNA (CS/Veh; 3.74 ± 0.42 RFC vs. Air/Veh: 2.25 ± 0.44 RFC, *p* < 0.05). When TSA was given intranasally prior to CS exposure (100 μg/ml, 50 μl/mouse), HO-1 expression was significantly inhibited (CS/Veh; 3.74 ± 0.42 RFC vs. CS/TSA: 2.25 ± 0.41 RFC, *p* < 0.05) (Fig. 2F).

### 3.4 HDAC2 knock-down decreases Nrf2 protein stability and activation

TSA strongly inhibits class I and class II HDAC activities but only HDAC2 is selectively down-regulated in the lung and alveolar macrophages from patients with COPD [11]. Selective inhibition of HDAC2 in BEAS2Bs was performed using short interference RNA with HDAC1 knock-down as a control. Transfection of siRNA targeting HDAC2 induced 45% reduction of protein level (Fig. 2G). H_2_O_2_ stimulation resulted in an increase in Nrf2 stability (2.4 ± 0.2-fold) in random oligonucleotide (RO) transfection controls whereas HDAC2 KD showed a significant reduction of both basal levels of Nrf2 (0.5 ± 0.0-fold; *p* < 0.05) and H_2_O_2_ stimulated Nrf2 (1.5 ± 0.3-fold; *p* < 0.05) compared with RO controls (Fig. 2G). By contrast, RNA interference of HDAC1 achieved 67% reduction of protein level, but HDAC1 KD did not alter basal or H_2_O_2_-stimulated Nrf2 stability (Fig. 2G).

### 3.5 HDAC2 protein correlates with Nrf2 protein expression in MDMs

**Fig. 3:**
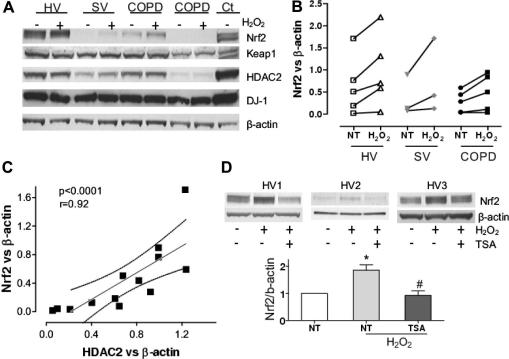
HDAC2 and Nrf2 expression correlation in monocyte-derived macrophages. (A) MDMs from a healthy volunteer (HV), a smoking volunteer (SV) and two COPD patients were stimulated with H_2_O_2_ (50 μM) for 30 min prior to whole-cell extraction and Western blotting. The expression of Nrf2, DJ-1, HDAC2, Keap1 and β-actin are shown. The whole cell extracts from unstimulated BEAS2B cells was used as positive control (Ct). (B) MDMs from five healthy volunteers (HV), three smoking volunteers (SV) and five COPD patients were stimulated with H_2_O_2_ (50 μM) for 30 min and expression of Nrf2 was normalized against β-actin. (C) Nrf2 and HDAC2 expression were correlated in all samples (n = 13). (D) MDMs from four healthy volunteers were stimulated with TSA (50 ng/ml) for 1.5 h prior exposure to H_2_O_2_ (50 μM) for 30 min and the expression of Nrf2 was normalized against β-actin. Values represent means ± SEM. NT (Non-treatment). ^∗^*p* < 0.01 for NT vs. H_2_O_2_ and ^#^*p* < 0.01 for H_2_O_2_ vs. TSA (H_2_O_2_).

Monocytes were collected from blood obtained from healthy subjects, smoking volunteers and COPD patients (Supplementary Table 1) and differentiated into MDMs. Cells were stimulated with H_2_O_2_ and Nrf2 protein was determined by Western blotting. As shown in Fig. 3A and B, basal Nrf2 expression was decreased in COPD compared to healthy volunteers although the difference was not significant (Fig. 3B). H_2_O_2_ increased Nrf2 protein levels in all subject and no significant differences in Nrf2 activation were observed between patients. However, a significant correlation was found between unstimulated Nrf2 expression and HDAC2 (*r* = 0.92, *p* < 0.0001, Fig. 3C). Additionally, TSA impaired Nrf2 increase in protein levels induced by H_2_O_2_ in MDMs from healthy volunteers (Fig. 3D), confirming results described in BEAS2B cells.

The expression levels of DJ-1 and Keap1 have previously been implicated in Nrf2 stability [9,10,21,22] but no differences in expression of DJ-1 or Keap1 were observed in COPD patients compared with healthy subjects (Fig. 3A). No correlation was also found between Nrf2 and DJ-1 (*r* = −0.45, *p* = 0.12) but the correlation with Keap1 almost reached significance (*r* = −0.54, *p* = 0.055), which has been previously observed in cancer cells [22].

### 3.6 HDAC2 is associated with Nrf2 and HDAC reduction causes acetylation of Nrf2

**Fig. 4:**
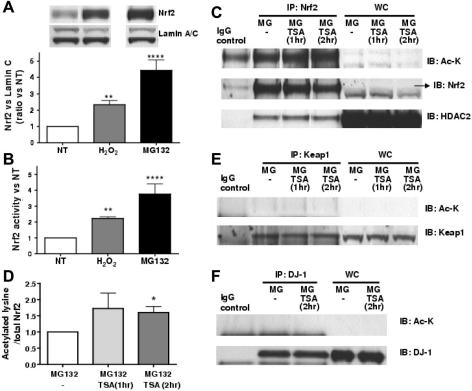
Nrf2 is acetylated with HDAC inhibition. (A) BEAS2B cells were treated with the proteasome inhibitor MG132 (5 μg/ml) for 1.5 h or with H_2_O_2_ (75 μM) for 30 min and Nrf2 and lamin C (control) were detected in nuclear extracts by Western blotting. (B) Nrf2 activity was determined in the same extracts using a TransAM kit, (*n* = 4), (^∗∗^*p* < 0.01 and ^∗∗∗∗^*p* < 0.0001 vs. NT [non-treatment]). (C) BEAS2B cells were stimulated with MG132 (5 μg/ml) for 2 h, with/without TSA (10 ng/ml for 1–2 h). Nrf2 was immunoprecipitated and acetylated-lysine (Ac-K) and HDAC2 were detected with the relevant-antibody. IP: immunoprecipitation, IB: immunoblot, WC: whole cell extracts. IgG: negative control for immunoprecipitation where no cellular lysates where used. The arrow (→) indicates the correct Nrf2 protein size in the whole cell extracts. (D) Band density of acetylated Nrf2 was determined and normalized to total Nrf2 expression (*n* = 3), ^∗^*p* < 0.05 vs. NT (non-treatment). Keap1 (E) and DJ-1 (F) were immunoprecipitaed from whole cell extracts under the same conditions used in (C).

In order to measure acetylation levels of Nrf2, high amount of the protein were obtained by treating BEAS2B cells with MG132, a proteasome inhibitor, preventing degradation of Nrf2. As shown in Fig. 4A, although H_2_O_2_ significantly stabilized Nrf2 protein in nuclei, MG132 further stabilized Nrf2 by approximately 4-fold. This suggests that H_2_O_2_ (75 μM) stabilized approximately 50% of total translated Nrf2. Nrf2 expression correlated with its activity (Fig. 4B). Nrf2 acetylation was measured after TSA treatment as shown in Fig. 4C. HDAC2 protein was detected in Nrf2 immunoprecipitates, suggesting that Nrf2 associates with HDAC2. This association was not altered in the presence of TSA (10 ng/ml) at 1 or 2 h stimulation prior whole-cell extraction. Nrf2 was also lysine-acetylated at baseline, and acetylation was increased in the presence of TSA at 1 and 2 h, reaching significance at 2 h (1.6 ± 0.2-fold; *p* < 0.05) (Fig. 4C and D). Immunoprecipitated Keap1 and DJ-1 in the presence of MG132 and/or TSA were not found to be acetylated (Fig. 4E and F).

## 4 Discussion

We have shown that in a COPD model, in which Class I and II HDACs are inhibited, Nrf2 protein is further acetylated and its stability is decreased along with its anti-oxidant potential which might contribute to COPD pathogenesis. We have also identified HDAC2 as one of the deacetylases likely to be responsible for this effect.

Oxidants, such as H_2_O_2_ target cysteine residues in Keap1 allowing Nrf2 to escape Cul3-mediated degradation [2]. In agreement with previous studies [23], we found that under basal conditions Nrf2 was localized in the nuclei and that activation by H_2_O_2_ increased its stability, ARE-driven activity and anti-oxidant gene expression.

Since HDAC and sirtuin activities have been shown to be reduced in COPD [11,24], we measured the effects of HDAC and sirtuin inhibition on Nrf2 protein and activity after stimulation with H_2_O_2_. Both activity and stability of Nrf2, at baseline and with H_2_O_2_ stimulation, were decreased after HDAC inhibition with TSA treatment but there was no effect with sirtuin inhibition with sirtinol, thus confirming that Class I and II HDACs are linked to Nrf2 stability. In fact, TSA was shown to reduce Nrf2 protein half-life both under oxidative stress and at baseline. In primary MDMs, TSA similarly impaired the H_2_O_2_ increase of Nrf2 protein levels as we have seen in BEAS2B cells. The anti-oxidant response after H_2_O_2_ stimulation, as measured by HO-1, was impaired in BEAS2B cells and this was confirmed in *in vivo* studies using mice. Moreover, in COPD, HDAC2 protein expression is known to be decreased [11] and knock-down of HDAC2, but not HDAC1, resulted in decreased Nrf2 stability. This was further confirmed in primary MDMs, where HDAC2 and Nrf2 expressions correlated, whereas no association was found with its inhibitor Keap1 and its positive regulator DJ-1. In agreement with our results, a recent study demonstrated that a mouse strain highly susceptible to emphysema, showed higher basal level of ROS concurrent with lower levels of Nrf2, HO-1 and HDAC2 compared to an emphysema insensitive-strain [25]. This suggests that HDAC2 is at least one of the Class I and II HDACs responsible for Nrf2 stability; however other HDACs apart from HDAC1 could also be involved.

Recent work has shown that Nrf2 acetylation at various residues in its Neh1 DNA-binding domain are required for full Nrf2 trans-activation but not for its stability [13]. BEAS2B cells stimulated with a proteasome inhibitor strongly stabilized and activated Nrf2 probably due to the saturation of the proteasome [26]. Interestingly, with proteasome inhibition, we found that Nrf2 was highly acetylated, in agreement with Sun et al. [13]*.* However, inhibition of HDAC activity by TSA showed that further acetylation of other residues in Nrf2 might be critical for Nrf2 protein stability. In the future, the residues contained in the Neh2 domain, the site of redox-sensitive ubiquitin conjugation, responsible for Keap1 interaction [27], should be analyzed as well as both Neh3 and Neh6 domains linked with protein stability [27,28]. Additionally, we have shown that HDAC2 co-immunoprecipitated with Nrf2, supporting the existence of a multi-molecular complex involving Nrf2 and HDAC2.

Collectively, our results demonstrate that HDAC2 stabilizes Nrf2 protein expression. We show for the first time that HDAC2 is associated with Nrf2 and prevents its degradation probably by deacetylation of lysine residues. As reduced HDAC2 activity is seen in COPD as a result of oxidative stress, this could reduce activity of Nrf2 so that an appropriate increase in antioxidant expression is blunted, thus increasing oxidative stress, which then further reduces HDAC2 activity in a vicious circle. Restoring HDAC2 expression and activity in COPD cells could prevent Nrf2 down-regulation by increased Nrf2 deacetylation resulting in the restoration of normal anti-oxidant defences.

## References

[1] Cho H.Y., Reddy S.P., DeBiase A., Yamamoto M., Kleeberger S.R. (2005). Gene expression profiling of NRF2-mediated protection against oxidative injury. Free Radical Biology and Medicine.

[2] Zhang D.D. (2006). Mechanistic studies of the Nrf2-Keap1 signaling pathway. Drug Metabolism Reviews.

[3] Kwak M.K., Wakabayashi N., Itoh K., Motohashi H., Yamamoto M., Kensler T.W. (2003). Modulation of gene expression by cancer chemopreventive dithiolethiones through the Keap1-Nrf2 pathway – identification of novel gene clusters for cell survival. Journal of Biological Chemistry.

[4] Lee J.M., Calkins M.J., Chan K.M., Kan Y.W., Johnson J.A. (2003). Identification of the NF-E2-related factor-2-dependent genes conferring protection against oxidative stress in primary cortical astrocytes using oligonucleotide microarray analysis. Journal of Biological Chemistry.

[5] Rangasamy T., Cho C.Y., Thimmulappa R.K., Zhen L.J., Srisuma S.S., Kensler T.W., Yamamoto M., Petrache I., Tuder R.M., Biswal S. (2004). Genetic ablation of Nrf2 enhances susceptibility to cigarette smoke-induced emphysema in mice. Journal of Clinical Investigation.

[6] Thimmulappa R.K., Mai K.H., Srisuma S., Kensler T.W., Yamamato M., Biswal S. (2002). Identification of Nrf2-regulated genes induced by the chemopreventive agent sulforaphane by oligonucleotide microarray. Cancer Research.

[7] Cho H.Y., Reddy S.P., Kleeberger S.R. (2006). Nrf2 defends the lung from oxidative stress. Antioxidants and Redox Signaling.

[8] Kobayashi M., Li L., Iwamoto N., Nakajima-Takagi Y., Kaneko H., Nakayama Y., Eguchi M., Wada Y., Kumagai Y., Yamamoto M. (2009). The Antioxidant defense system Keap1-Nrf2 comprises a multiple sensing mechanism for responding to a wide range of chemical compounds. Molecular and Cellular Biology.

[9] Goven D., Boutten A., Lecon-Malas V., Marchal-Somme J., Amara N., Crestani B., Fournier M., Leseche G., Soler P., Boczkowski J., Bonay M. (2008). Altered Nrf2/Keap1-Bach1 equilibrium in pulmonary emphysema. Thorax.

[10] Malhotra D., Thimmulappa R., Navas-Acien A., Sandford A., Elliott M., Singh A., Chen L.N., Zhuang X.X., Hogg J., Pare P., Tuder R.M., Biswal S. (2008). Decline in NRF2-regulated antioxidants in chronic obstructive pulmonary disease lungs due to loss of its positive regulator, DJ-1. American Journal of Respiratory and Critical Care Medicine.

[11] Ito K., Ito M., Elliott W.M., Cosio B., Caramori G., Kon O.M., Barczyk A., Hayashi S., Adcock I.M., Hogg J.C., Barnes P.J. (2005). Decreased histone deacetylase activity in chronic obstructive pulmonary disease. New England Journal of Medicine.

[12] Ito K., Yamamura S., Essilfie-Quaye S., Cosio B., Ito M., Barnes P.J., Adcock I.M. (2006). Histone deacetylase 2-mediated deacetylation of the glucocorticoid receptor enables NF-kappa B suppression. Journal of Experimental Medicine.

[13] Sun Z., Chin Y.E., Zhang D.D. (2009). Acetylation of Nrf2 by p300/CBP augments promoter-specific DNA Binding of Nrf2 during the antioxidant response. Molecular and Cellular Biology.

[14] Ito K., Hanazawa T., Tomita K., Barnes P.J., Adcock I.M. (2004). Oxidative stress reduces histone deacetylase 2 activity and enhances IL-8 gene expression: role of tyrosine nitration. Biochemical and Biophysical Research Communications.

[15] Kruger M., Kratchmarova I., Blagoev B., Tseng Y.H., Kahn C.R., Mann M. (2008). Dissection of the insulin signaling pathway via quantitative phosphoproteomics. Proceedings of the National Academy of Sciences of the United States of America.

[16] Sussan T.E., Rangasamy T., Blake D.J., Malhotra D., El Haddad H., Bedja D., Yates M.S., Kombairaju P., Yamamoto M., Liby K.T., Sporn M.B., Gabrielson K.L., Champion H.C., Tuder R.M., Kensler T.W., Biswal S. (2009). Targeting Nrf2 with the triterpenoid CDDO-imidazolide attenuates cigarette smoke-induced emphysema and cardiac dysfunction in mice. Proceedings of the National Academy of Sciences of the United States of America.

[17] Kobayashi A., Kang M.I., Okawa H., Ohtsuji M., Zenke Y., Chiba T., Igarashi K., Yamamoto M. (2004). Oxidative stress sensor Keap1 functions as an adaptor for Cul3-based E3 ligase to regulate for proteasomal degradation of Nrf2. Molecular and Cellular Biology.

[18] Itoh M., Adachi M., Yasui H., Takekawa M., Tanaka H., Imai K. (2002). Nuclear export of glucocorticoid receptor is enhanced by c-Jun N-terminal kinase-mediated phosphorylation. Molecular Endocrinology.

[19] Jain A.K., Bloom D.A., Jaiswal A.K. (2005). Nuclear import and export signals in control of Nrf2. Journal of Biological Chemistry.

[20] McMahon M., Itoh K., Yamamoto M., Hayes J.D. (2003). Keap1-dependent proteasomal degradation of transcription factor Nrf2 contributes to the negative regulation of antioxidant response element-driven gene expression. Journal of Biological Chemistry.

[21] Clements C.M., McNally R.S., Conti B.J., Mak T.W., Ting J.P.Y. (2006). DJ-1, a cancer- and Parkinson’s disease-associated protein, stabilizes the antioxidant transcriptional master regulator Nrf2. Proceedings of the National Academy of Sciences of the United States of America.

[22] Singh A., Misra V., Thimmulappa R.K., Lee H., Ames S., Hoque M.O., Herman J.G., Baylin S.B., Sidransky D., Gabrielson E., Brock M.V., Biswal S. (2006). Dysfunctional KEAP1-NRF2 interaction in non-small-cell lung cancer. Plos Medicine.

[23] Nguyen T., Sherratt P.J., Nioi P., Yang C.S., Pickett C.B. (2005). Nrf2 controls constitutive and inducible expression of ARE-driven genes through a dynamic pathway involving nucleocytoplasmic shuttling by Keap1. Journal of Biological Chemistry.

[24] Nakamaru Y., Vuppusetty C., Wada H., Milne J.C., Ito M., Rossios C., Elliot M., Hogg J., Kharitonov S., Goto H., Bemis J.E., Elliott P., Barnes P.J., Ito K. (2009). A protein deacetylase SIRT1 is a negative regulator of metalloproteinase-9. FASEB Journal.

[25] Vecchio D., Arezzini B., Pecorelli A., Valacchi G., Martorana P.A., Gardi C. (2010). Reactivity of Mouse Alveolar Macrophages to Cigarette Smoke is train Dependent. American Journal Physiology Lung Cell Molecular Physiology.

[26] Stewart D., Killeen E., Naquin R., Alam S., Alam J. (2003). Degradation of transcription factor Nrf2 via the ubiquitin-proteasome pathway and stabilization by cadmium. Journal of Biological Chemistry.

[27] McMahon M., Thomas N., Itoh K., Yamamoto M., Hayes J.D. (2004). Redox-regulated turnover of Nrf2 is determined by at least two separate protein domains, the redox-sensitive Neh2 degron and the redox-insensitive Neh6 degron. Journal of Biological Chemistry.

[28] Nioi P., Nguyen T., Sherratt P.J., Pickett C.B. (2005). The carboxy-terminal Neh3 domain of Nrf2 is required for transcriptional activation. Molecular and Cellular Biology.

