# Supplementary figures and images for: Decreased histone deacetylase 2 impairs Nrf2 activation by oxidative stress
Source: Biochem Biophys Res Commun. 2011 Mar 11;406(2):292–8. doi: 10.1016/j.bbrc.2011.02.035 (PMC3061319; doi:10.1016/j.bbrc.2011.02.035)

**Supplementary Figure 1**

**
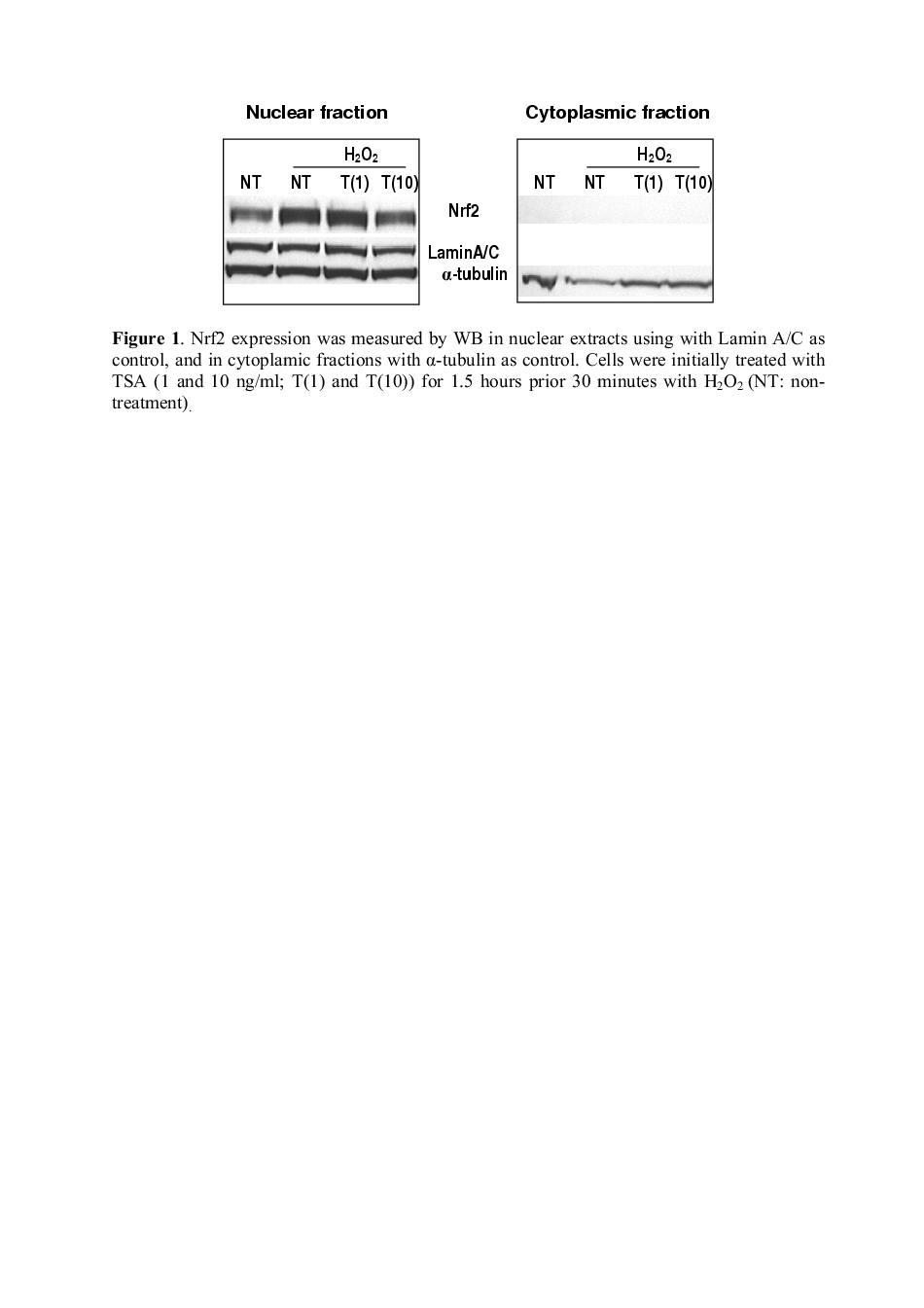
**

**Supplementary Figure 2**

**
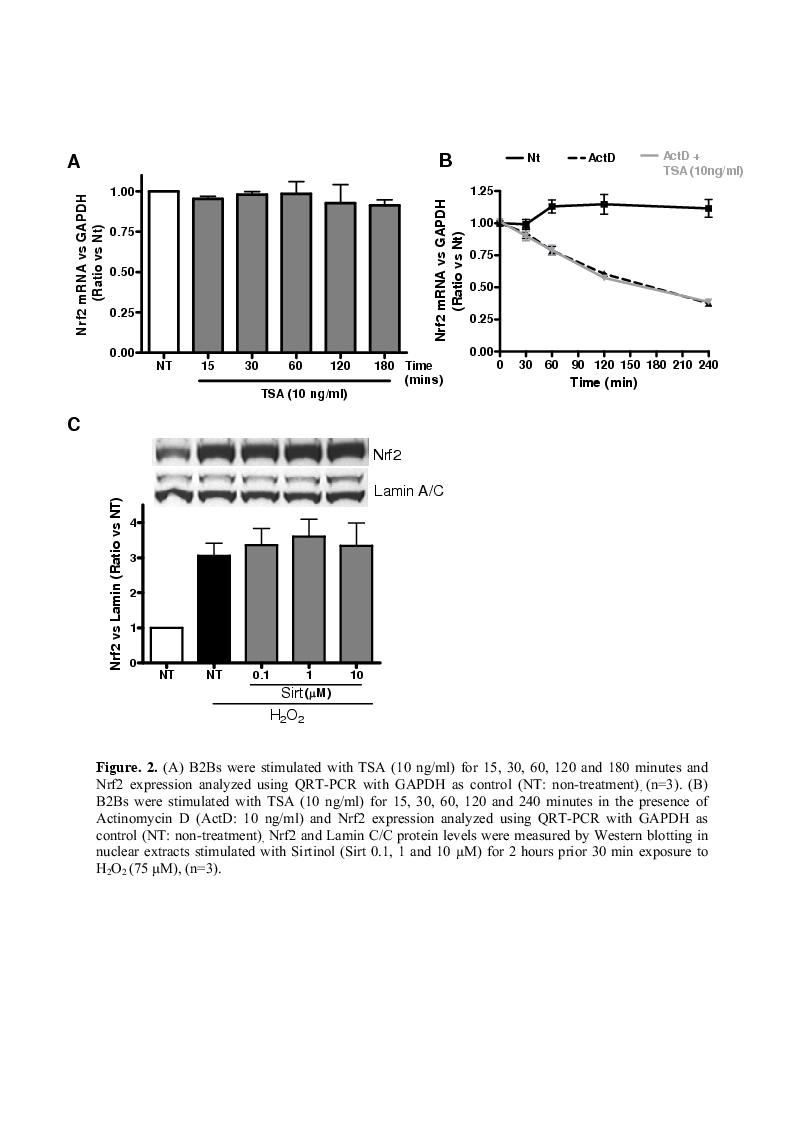
**

**Supplementary Table 1**

**
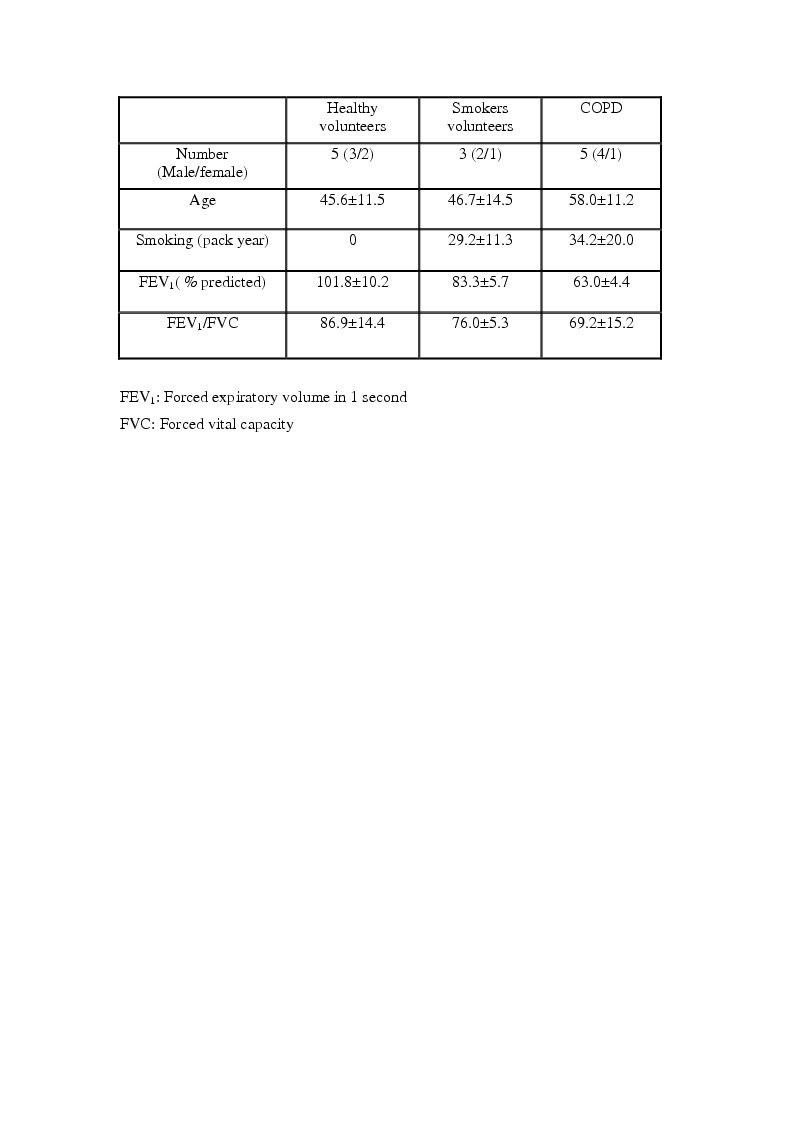
**

Supplement: Supplementary data 1 [file mmc1.doc]
